# An Emerging Infectious Yeast to Watch: *Cryptococcus gattii* Species Complex

**DOI:** 10.3390/jof11120858

**Published:** 2025-12-02

**Authors:** Samantha N. Peltak, Tomoko Y. Steen

**Affiliations:** Department of Microbiology and Immunology, Georgetown University School of Medicine, 3900 Reservoir Rd. NW, Washington, DC 20007, USA; snp64@georgetown.edu

**Keywords:** infectious disease, public health, mycology, cryptococcosis, *Cryptococcus gattii*, *Cryptococcus gattii* species complex

## Abstract

CGSC is a pathogenic basidiomycetous yeast of increasing public health concern due to its ability to cause life-threatening infections in both immunocompromised and immunocompetent individuals. *C. gattii* species complex (CGSC) is acquired via environmental exposure, particularly through the inhalation of spores from trees, soil, and decaying wood. Infections often manifest in the pulmonary or central nervous system, both of which are associated with high morbidity and mortality. Compounding this threat is the pathogen’s expanding geographic range, facilitated in part by climate change, and the limited effectiveness of antifungal therapies that are available. Genetic diversity among molecular types (VGI–VGVI) contributes to variable antifungal susceptibility, further complicating treatment. Knowledge of risk factors for the CGSC are limited. Despite its rising global footprint and potential for severe diseases, CGSC remains underreported, with surveillance gaps even in endemic regions. This review highlights the pathogen’s epidemiology, risk factors, clinical impact, and therapeutic challenges, arguing for changes in policy that would increase reporting efforts worldwide. Improved surveillance, public health education, and antifungal research are critical to curbing the growing burden of CGSC infections worldwide.

## 1. Introduction

Throughout history, infectious diseases have impacted human civilization. These fall into two categories: emerging and re-emerging diseases [1]. Emerging diseases are classified as those that are newly recognized in a population, increasing in incidence, or expanding in geographic range [2]. Re-emerging diseases are established illnesses that can resurface due to a change in condition, including both to the environment and the host demographic [1]. According to the International Society for Infectious Diseases (ISID), global estimates suggest that approximately 6.5 million people acquire invasive fungal infections yearly and over half of those infections result in mortality [3].

One of these emerging pathogens is *Cryptococcus gattii* species complex (CGSC)*,* both a primary and an opportunistic yeast, which is one of the causative agents of cryptococcosis [4]. The first reported human infection of CGSC took place in 1896 in France but it was not named until 1970; when researchers described a distinct group of elliptical shaped yeast cells caused by a cryptococcal strain isolated from a pediatric African patient with leukemia, they called the isolate *Cryptococcus neoformans* var. *gattii* [5,6]. Researchers performed a cultural analysis on the sample found in France to confirm it was the same clinical isolate they were working with that was seen in the African patient [6]. After this finding, evidence attesting to differences in morphology, biochemistry, ecology, and clinical features between *C. neoformans* and *C. gattii* were found. The two pathogens fulfilled the criteria to be considered two separate species, which was officially noted in 2002 [7].

Debate over nomenclature did not stop once these two species were separated, however. Within the species *C. gattii*, there are six molecular types, known as VGI–VI, which will be discussed later in this review. These molecular types have been proposed as distinct species but are collectively known as *C. gattii* species complex. Currently, the CGSC includes VGI–IV, as VGV was discovered within the last 6 years and VGVI is exceedingly rare. The debate comes from genetic evidence displaying distinct evolutionary divisions within the molecular types, which has some experts calling for formal name changes [8]. However, some researchers argue for maintaining the species complex notation for nomenclature stability [9]. In this review, going forward, *C. gattii* will be noted as CGSC unless specific molecular types are being discussed.

The biggest difference between *C. gattii* species complex and *C. neoformans* is that while the latter has been seen to infect primarily immunocompromised individuals, the former infects both immunocompromised and immunocompetent individuals and is more frequently associated with healthy persons, those with no identified diseases or immune deficits [4]. CGSC has slowly broadened the geographical range and environmental niches it has resided in for the last thirty years [10,11]. Thus, with an increase in susceptible hosts, it has become a global health burden. To add to this problem, there are limited fungal treatment options, in part because fungi and animals share many conserved signaling cascades [10]. Currently, CGSC is seen significantly less frequently than the other agent of cryptococcosis, *C. neoformans*, though due to its ability to infect healthy individuals and its increased presence due to climate change, it should be surveilled just as vigilantly [4,12]. The World Health Organization (WHO) labeled CGSC a medium-risk pathogen in 2022 [13]. The WHO report, along with the pathogen’s movement into new geographical regions, limited treatment options, and antimicrobial resistance provide evidence as to why there needs to be reporting standards put into place for policy makers, given the global health risks this pathogen increasingly represents.

## 2. Ecology

*C. gattii* is a sexually dimorphic yeast that grows via asexual budding in the environment but can undergo sexual replication as well, growing filamentous hyphal structures that provide nutrients and allow for growth and reproduction. At the end of these hyphal structures there are buds called basidiospores, which are oval or cylindrical shaped [5]. The CGSC can be found in approximately 50 species of trees, decaying wood, contaminated air, and soil [11]. The species list is located in Table 1. The specific niche information for CGSC is important when assessing risk factors, understanding epidemiology, and to aid in the development of effective prevention strategies. The primary mode of transmission is environmental exposure and inhalation of the spores or desiccated yeast cells during dry months, typically ranging from late spring to early fall. Once inhaled, the yeast travels through the respiratory system to the lungs. This initial infection can then disseminate to other locations such as the central nervous system (CNS) and other organs [10].

## 3. Geographic Expansion

**Figure 1:**
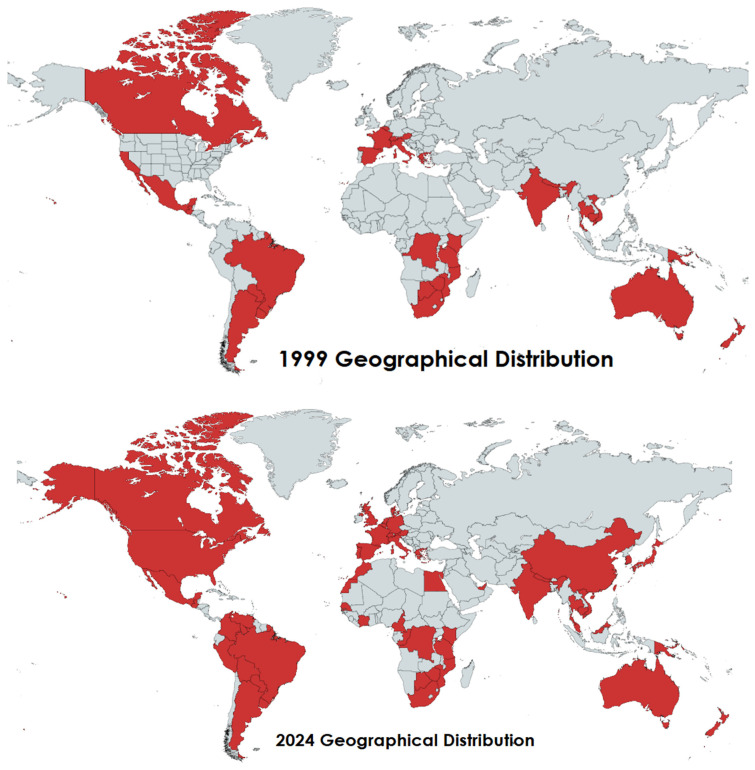
Maps comparing geographic growth of *Cryptococcus gattii* species complex in 1999 and 2024 [5,12,14,22,23,24]. (Created by the authors with mapchart.net). Note. Geographical distribution increase in CGSC cases from 1999 to 2024. The yeast had only been known to inhabit tropical and subtropical regions until 1999, when it was found in Vancouver Canada. Since then, it has had a great expansion into the U.S., Africa, and parts of Asia. Representation is based on reporting efforts, which may be a limitation of this geographic map.

The *C. gattii* species complex is seen in Figure 1 expanding its geographical range from 1999 to 2021 [5,12,14,22,23,24]. To construct the data for the 1999 map, several reports were taken into consideration. A study from 1997 investigated 696 samples and found evidence of CGSC in India for the first time [22]. A report from 1984 used data from 628 clinical isolates and found high prevalence of CGSC in Australia, Brazil, Cambodia, Hawaii, Southern California, Mexico, Paraguay, Thailand, Vietnam, Nepal, and countries in Central Africa [23]. A 2011 study found that there were new outbreaks in regions of the U.S., Canada, and British Columbia between 2004 and 2009 by analyzing 52 patient reports [5]. A 2009 study compared 489 samples found in trees with those reported in 2001 and reinforced previous notions of the prevalence in Argentina [14]. A study investigating the impact of global warming showed 2020–2029 projections predicting further spread into northern and inland regions of Europe due to rising temperatures. This was predicted using ecological niche modeling with data from 1980 to 2019. Researchers used temperature and precipitation data to model the environmental conditions where CGSC thrived, which provides evidence of the yeast’s adaptation and spread into new regions. The study found a slight expansion of the CGSC niche in Europe from 1980 to 1999, and again from 2000 to 2009 toward France and Northern Europe. From 2010 to 2019, the CGSC further expanded to include Spain, Southern France, Italy, Croatia, parts of Greece, and Turkey [12]. This study highlights how the emergence of CGSC in more temperate areas indicates a shift in the geographic niche, which is consistent with global warming trends. This, in turn, increases the chances of human exposure in places the yeast previously could not survive, linking climate and environmental health to the risk of an infectious disease outbreak. Another article took data from 604 reports of cases from 1970 to 2021 and included both animal and human reports of CGSC [24]. These three reports were used to construct the 2024 map.

## 4. Molecular Types

The microbe was previously thought to have four major molecular types, *Variety gattii* (VG) one through four; however, in 2019 a new lineage named VGV was discovered in Zambia, Africa. VGV varies from the other four types by approximately 0.75 million single nucleotide polymorphisms (SNPs), which are genomic variations at a single position in the DNA sequence. These differences allow it to be classified as a novel variety. Not much is known about its geographical prevalence or clinical manifestation [21]. There have been few cited cases of a sixth molecular type, VGVI, which is thought to be delineated from VGIII. VGVI is rare but there have been some clinical human cases from Mexico, South America, and the American Southwest [25,26].

**Figure 2:**
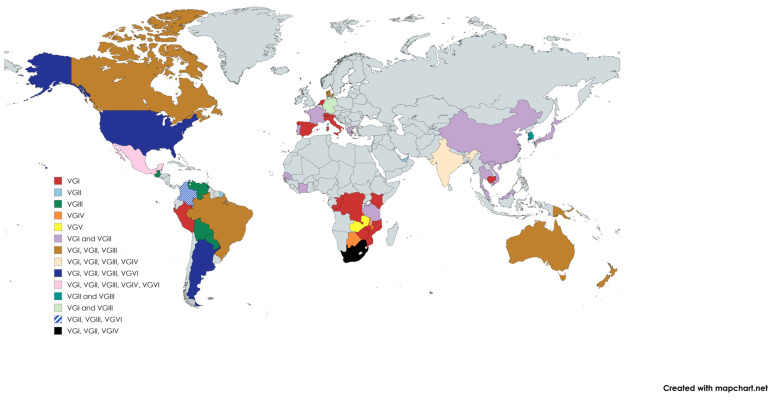
A 2024 geographical distribution of the six molecular types [24] (created by the authors). Note. The distribution of the six molecular types. Though all types can be seen around the world, it can be noted that VGII and VGIII are found more often in warmer climates, while VGIV is found in hotter climates like those seen in Africa, South America, and India. VGV was discovered in Zambia, Africa, and has not been seen in another location thus far. VGVI has been found in the American Southwest, some parts of South America, and was previously noted in Mexico in the 1960s.

The molecular types are attributed to different geographical regions and clinical features, as seen in Figure 2 and Table 2 [24]. Some countries see more than one molecular type while others only have predominately one, these groupings are made clear in Figure 2. VGIV and VGIII are prevalent in tropical and subtropical regions such as Africa, Southern California, and South America [10]. VGII is common in North and South America and was responsible for an outbreak on Vancouver Island from 1999 to 2007. VGI is most frequently seen in Asia, Europe, and Australia. The latter has recognized CGSC as an endemic pathogen since before 1999 [27].

Clinically, VGI and VGII have been found to be the two subtypes to cause the most infections, as well as being the two that infect immunocompetent individuals. These two molecular types are in most of the countries affected by CGSC, which can be seen in Figure 2 [10]. VGII has been attributed as being one of the most antimicrobial resistant, with subtypes of A, B, and C [28]. Both VGII and VGIII have been considered the most virulent strains in drosophila models [29,30]. Murine models have not shown the same results for VGII and have even seen variation in virulence among different subtypes [31,32]. Based on a review of existing literature, it is unclear if comparative virulence analysis has been performed between VGIII and other subtypes in murine models, although variations among subtypes have been noted [20]. Molecular types VGIII and VGIV are associated more strongly with immunocompromised individuals [10].

## 5. Risk Factors

While risk factors are not well defined, it is understood that molecular type plays a role. VGI and VGII are associated more frequently with those without clear medical risk, while VGIII and VGIV are seen in those who are immunocompromised, such as HIV/AIDs patients and those who have autoimmune diseases that use steroids [4]. A 2014 study determined that individuals who had the granulocyte-macrophage colony-stimulating factor (GM-CSF) neutralizing antibodies had a higher risk of cryptococcosis infection, despite being immunocompetent. GM-CSF is a cytokine, which is a type of growth factor that stimulates the production of white blood cells such as granulocytes and macrophages. GM-CSF is critical in alveolar macrophage differentiation, as it promotes the survival, proliferation, and maturation of these cells. GM-GSF is also crucial in the development of normal innate immune function of the lung, which is where it would come in contact with the pathogenic agent. While there is an association between the GM-CSF antibodies and increased risk for disease due to *Cryptococcus* spp., it is important to note that they are not specific to the pathogen CGSC and more studies would need to be conducted to form a stronger link [4,27]. Though there are no strongly associated risk factors, patient history should be taken to determine if they had contact with the animal *Phascolarctos cinereus* or any of the tree types that are strongly associated with CGSC, referenced in Table 1 [33].

## 6. Epidemiology

Since 1970, there has been a steady increase in cases of cryptococcosis around the globe. The real number of cases caused by CGSC is unknown because until the early 2000s, cases of cryptococcosis were not sampled and sequenced to differentiate between CGSC and *C. neoformans* [33]. This is an important limitation to remember when analyzing Figure 1, as the map of 1999 is only as accurate as the limitations of the time permit. A particularly interesting outbreak from 1999 to 2007 occurred on Vancouver Island in Canada, where 218 people acquired the infection [23]. These cases were specifically noteworthy because up until this point, this yeast was primarily found in tropical, subtropical, and some temperate areas such as Australia, Papua New Guinea, and Brazil [34]. A similar type of unseen expansion occurred again in the Pacific Northwest of the United States around 2004 [35].

In a 2023 study, 426 patients from 46 hospitals across New Zealand and Australia had confirmed cryptococcosis cases and were HIV negative. Out of 87 confirmed CGSC cases, 82 were HIV negative, which was 94% of the total. Of the 426 HIV negative patients in total, 259 did have a predisposed immunocompromising condition including cancer, organ transplant recipient, rheumatoid arthritis, or multiple sclerosis, while 167 had no identifiable condition. Only 37% of the 167 immunocompetent individuals were identified to have *C. neoformans* infections. The presence of an underlying condition was significantly associated with *C. neoformans*, 72.8%, compared to CGSC, 26.8%. This led to the study determining that the presence of an immunocompromising disorder was more often linked with *C. neoformans* over CGSC. The same study found the likelihood of male patients contracting cryptococcosis when compared to female patients was in a 2:1 ratio [36].

As for surveillance and reporting efforts, a lot is left to be desired, especially in countries that have higher rates of exposure, such as the North America, parts of South America, and Papua New Guinea [34,37,38,39,40,41,42]. In the U.S., CGSC disease reporting is only required in Washington state and Oregon. CGSC infections are not nationally notifiable despite it being one of the countries that considers the yeast to be endemic in some areas [37]. Canada does not have country-wide reporting requirements for CGSC either, but in certain territories, such as British Columbia and Alberta, positive test results must be conveyed to the government [38,39]. A similar system is seen in South America, where surveillance is voluntary. The country has no universal reporting requirements, despite the pathogen being endemic in Brazil and cryptococcosis being endemic in Colombia [40,41]. Australia, a country that has had CGSC established as an endemic pathogen for over twenty-five years, does not have the yeast listed on its National Notifiable Diseases Surveillance System [42]. Lastly, while CGSC is established as an endemic pathogen in Papua New Guinea, it is undefined whether there are reporting efforts being made, as these concerns have not been addressed in existing literature.

This lack of required public health surveillance systems contributes to the challenges in accurately assessing the true incidence and prevalence of CGSC infections. Fungal infections that are reported on, including coccidioidomycosis infections, have not had a noticeable reduction in cases either. Coccidioidomycosis infection cases are one of two fungal diseases that are nationally notifiable in the U.S. and have continued to increase in case counts. However, it should be noted that while cases rise, recommended prevention and infection control strategies have been successful in preventing spread. Other factors that contribute to a perceived increase in cases are improved awareness and diagnostic practices [43]. Coccidioidomycosis and CGSC are both inhaled from the environment and cause similar infections. Perhaps with policy changes that mandate reporting efforts in conjunction with preventative education materials listed on a public health website like those that have worked with Coccidioidomycosis infections, the risk of severe CGSC infection could be reduced.

## 7. Clinical Presentation

Cryptococcosis, the fungal infection caused by *Cryptococcus* species, can manifest as both CNS and pulmonary diseases [23,33]. Since *Cryptococcus* species are typically acquired through inhalation, pulmonary involvement is frequent, with CGSC infections leading to the formation of pulmonary nodules or mass lesions, called cryptococcomas, following prolonged exposure in the lungs [44]. However, the most severe complications and many fatalities are primarily linked to meningoencephalitis, which is inflammation of the meninges and brain tissue [5]. CNS cryptococcosis can take the form of meningitis (inflammation of the meninges, which protect the brain and spinal cord), clustered cysts, meningoencephalitis, or choroid plexitis (inflammation of the choroid plexus, which makes cerebral spinal fluid) [45]. A differentiating feature between the two causative agents of this disease is that CGSC infections tend to see yeast accumulation in the lungs and the brain, which cause lesions, while in *C. neoformans* this phenomenon is less frequent and the lesions are often smaller than those of CGSC in those who are immunocompromised [5,33]. Globally, an estimated 194,000 people develop this complication each year, with approximately 147,000 resulting in fatalities. This equates to a mortality rate of roughly 76% [3]. CGSC causes between 11–33% of invasive cryptococcus globally and is associated with a 10–25% fatality rate [46].

Many factors can help reduce this death toll, including a swift and accurate diagnosis; unfortunately, the incubation period for CGSC infections is quite long, with the median time being six to seven months [47]. This impacts the diagnostic time and subsequent patient outcomes since patients are slow to seek medical attention. While fungal culture is a reliable method, it can take several days to grow and identify the source, so current guidelines recommend any patient presenting with compatible symptomology and microbiology should be considered to have cryptococcosis [48]. In addition to fungal culture and antigen testing, diagnosis is made through chest and brain imaging as cryptococcomas may be observed. Respiratory or cerebral spinal fluid samples are used in diagnostics as the infection congregates within the brain or lungs in the body, so there is a higher likelihood of obtaining a positive diagnosis from these samples [27]. In order to confirm a CGSC infection, the yeast recovered must be genotyped from culture [23]. Typically, CGSC infections respond more slowly to antifungal drugs than *C. neoformans* and antifungal susceptibility varies among genotypes [5]. Not only have they been seen to respond slower, CGSC has also been found to have less antimicrobial susceptibility than *C. neoformans* [28].

## 8. Antimicrobial Susceptibility

Amphotericin B has been the primary treatment for these infections as recently as 2021, as resistance has been rarely seen. Other important antifungal options include fluconazole and flucytosine. Higher resistance to antifungal drugs is seen in the VGII strains A, B, and C, particularly in patients infected with rare CNS lesions called cryptococcomas. If these cryptococcomas do not have a reduction in size after four weeks, surgical intervention is recommended due to the potential complications associated with increased size of mass lesions, neurological symptoms, and development of antifungal resistance. A Japanese study saw success with use of voriconazole as a non-surgical alternative to remove these lesions [28]. While this one study saw success with the antifungal medication, more studies would need to be conducted to confirm its efficacy in treating these lesions.

Variation in antimicrobial susceptibility has been seen among the molecular types VGI–VGVI. VGI was seen to be more susceptible to azoles and flucytosine when compared to VGII. However, VGI and VGII were less susceptible than VGIII to azoles in some studies [49]. A Colombian study in 2023 found that there was reduced susceptibility to azoles in CGSC species due to a mutation resulting in the substitution of R258L at the recognition site of ERG11 [50]. This is the only study which has found this potential source of increased antimicrobial resistance. Variation in susceptibility among different studies has led to a lack of clinical breakpoints. The absence of these breakpoints means that while there is minimum inhibitory concentration (MIC) data that can guide treatment choices, they are not definitive predictors in all clinical case outcomes [51]. This is because MIC data alone provides limited information, as in vitro studies do not consider human response factors or dosing variables, which are both aspects that are crucial therapeutic success [52]. This disparity among studies and lack of data on definitive clinical breakpoints to recognize antifungal susceptibility prevent more effective treatments from being discovered.

The WHO Fungal Priority Pathogen List considered CGSC a medium priority due to the lack of effective treatment and its predictors of infection [13]. As of 2022, there have been no trials for novel therapeutics [46]. Azoles work by interacting with lanosterol 14-α-demethylase, which is encoded by the gene ERG11. Perhaps future research could use these mutated ERG11 sites to create a more susceptible target for antifungal drugs.

## 9. Conclusions

While CGSC may not seem like the traditional candidate for an emerging infectious disease agent, this does not diminish its significance as such. CGSC is a formidable fungal pathogen whose significance in global public health due to its ability to infect immunocompetent individuals, expanding environmental range, and varying susceptibility to antifungal therapies calls for increased surveillance for the yeast. Unlike many infectious agents, CGSC is not transmitted between humans, yet its presence in diverse environmental reservoirs, which include over 50 species of trees, creates widespread and unpredictable exposure risks. Climate change, land-use alterations, and globalization have accelerated its geographic spread, as evidenced by outbreaks in previously unaffected regions like the Pacific Northwest. The diversity of its molecular types (VGI–VGVI), each with variable pathogenicity and antifungal susceptibility, complicates both diagnosis and treatment.

Infections frequently present severe pulmonary or central nervous system disease, with high mortality rates and limited treatment efficacy. Compounding this threat are delayed incubation periods, gaps in public health surveillance, and an absence of effective therapeutics targeting resistant strains or novel molecular variants.

Despite its rising incidence and the recognition as a medium-priority pathogen by the WHO, surveillance remains inconsistent and grossly inadequate. Most countries, including those with endemic CGSC, such as Australia, Canada, parts of the United States, and South America, lack mandatory reporting requirements. As a result, infection data is fragmented, true case numbers remain underestimated, and public health agencies are unable to effectively track environmental reservoirs or predict potential outbreaks.

To address these challenges, international policy must prioritize standardized global policy and mandate reporting of CGSC infections. Enhanced monitoring would allow for earlier outbreak detection, more accurate epidemiological data, and greatly improve the understanding of antifungal resistance patterns. As a pathogen that straddles the environmental and clinical domains, CGSC exemplifies the importance of a multidisciplinary approach. Addressing its threat requires a coordinated effort across disciplines from microbiology and immunology to environmental science and public health policy.

## Figures and Tables

**Table 1:** Tree species representing a niche for *Cryptococcus gattii* species complex and their associated locations (created by the authors).

Location	Species (Common Name)	
Argentina [14]	Acacia Visco Deodar Cedar Eucalyptus species	Mediterranean Cypress Smooth leaf Elm Tipu
Australia [15]	Blackbutt Blakely’s Red Gum Darwin Stringybark Eucalyptus species Flood Gum Forest Red Gum Paperbark	Poplar Box Smooth Bark Apple Swamp Mahogany Tallowwood Tuart Tree Turpentine
Brazil [16]	Eucalyptus species Ficus Species	Pink Shower Tree Pottery Tree
Canada [17]	Alder species Cedar species Douglas Fir Grand fir	Pine species Spruce species Western Hemlock Western Red Cedar
Colombia [18]	Almond tree Bogota Croton Coussapoa species Ficus species Funcks Croton/Funks Caper	Green Wattle Monterey Cyprus Red River Gum Rubber Savanna Tasmanian Blue Gum
India [19]	Arjuna Bullet wood/Indian Madlar Tree Eucalyptus species Golden Shower Indian Mast Tree Java Plum Lemon-scented Gum	Manila Tamarind Margosa Neem Tamarind Thorn tree Mango
United States [20]	American Sweetgum Canary Island Pine	Pohutukawa Red River Gum
Zambia [21]	Southern Tree Hyrax	

Note. Common names of species that are inhabited by *C. gattii* species complex. Similar or exact species can be seen among the eleven different countries.

**Table 2:** Descriptions of the six major molecular types of *Cryptococcus gattii* (created by the authors).

Molecular Type	Clinical Features	Distribution
VGI	Most common in humans and animals, highly clonal	High distribution in Australia, all over the world
VGII	Highly virulent genotypes identified	Vancouver Island Outbreak, Asia
VGIII	Associated with immunocompromised patients, highly virulent	High levels observed in Southern California, Mexico, and South America
VGIV	Associated with immunocompromised patients	Rare but found in Africa, India, and South America
VGV	Not enough data	Zambia Africa
VGVI	Not enough data	American Southwest, Mexico (1960)

Note. Descriptions for each of the six molecular types. VGV and VGVI have not had enough data collected to determine clinical features.

## Data Availability

No new data were created or analyzed in this study. Data sharing is not applicable to this article.
